# Draft Genome Sequence of *Frankia* sp. Strain BMG5.12, a Nitrogen-Fixing Actinobacterium Isolated from Tunisian Soils

**DOI:** 10.1128/genomeA.00468-13

**Published:** 2013-07-11

**Authors:** Imen Nouioui, Nicholas Beauchemin, Michael N. Cantor, Amy Chen, J. Chris Detter, Teal Furnholm, Faten Ghodhbane-Gtari, Lynne Goodwin, Maher Gtari, Cliff Han, James Han, Marcel Huntemann, Susan Xinyu Hua, Natalia Ivanova, Nikos Kyrpides, Victor Markowitz, Kostas Mavrommatis, Natalia Mikhailova, Henrik P. Nordberg, Galina Ovchinnikova, Ioanna Pagani, Amrita Pati, Arnab Sen, Saubashya Sur, Ernest Szeto, Subarna Thakur, Luis Wall, Chia-Lin Wei, Tanja Woyke, Louis S. Tisa

**Affiliations:** University of Tunis-El Manar, Tunis, Tunisiaa; University of New Hampshire, Durham, New Hampshire, USAb; Los Alamos National Laboratory, Los Alamos, New Mexico, USAc; DOE Joint Genome Institute, Walnut Creek, California, USAd; University of North Bengal, Siliguri, Indiae; University of Quilmes, Buenos Aires, Argentinaf

## Abstract

Members of the actinomycete genus *Frankia* form a nitrogen-fixing symbiosis with 8 different families of actinorhizal plants. We report a draft genome sequence for *Frankia* sp. strain BMG5.12, a nitrogen-fixing actinobacterium isolated from Tunisian soils with the ability to infect *Elaeagnus angustifolia* and *Myrica gale*.

## GENOME ANNOUNCEMENT

Among the *Actinobacteria*, the genus *Frankia* is well known for its facultative lifestyle as a plant symbiont of dicotyledonous plants and as a free-living soil dweller (1–3). The symbiosis allows actinorhizal plants to colonize harsh environmental terrains under diverse ecological conditions. Based on several criteria, including the 16S rRNA gene (4), *glnII* (5, 6), *gyrB* (6), and a 16S-23S rRNA intertranscribed spacer region (7), phylogenetic analysis has identified four distinct clusters among the *Frankia* strains. Genomes for representatives from each of these clusters have been sequenced (8–12) and have provided vital baseline information for genomic approaches toward understanding these novel bacteria.

Members of cluster III *Frankia* strains are considered broad-host-range symbionts and are known to associate with five plant families (*Betulaceae*, *Myricaceae*, *Elaeagnaceae*, *Rhamnaceae*, and *Casuarinaceae*). *Frankia* sp. strain BMG5.12 was chosen for sequencing as another cluster III representative with broad-host-range properties. Strain BMG 5.12 was isolated from Tunisian soils that were devoid of *Elaeagnus* plants and it was found to infect these host plants (13). Strain BMG5.12 was sequenced to find information about the potential ecological roles of the *Frankia* strains and their interactions with actinorhizal plants. As a representative from a harsh dry climate, the *Frankia* sp. BMG5.12 genome may provide insight on its ability to adapt to these arid hot soils that have high salinity.

The draft genome sequence of *Frankia* sp. BMG5.12 was generated at the Department of Energy (DOE) Joint Genome Institute (JGI) using Illumina technology (14). An Illumina standard shotgun library was constructed and sequenced using the Illumina HiSeq 2000 platform, which generated 9,945,424 reads totaling 1,491.8 Mbp. All techniques for DNA isolation, library construction, and sequencing were performed according to JGI standards and protocols (http://www.jgi.doe.gov). The Illumina sequence data were assembled using Velvet (version 1.1.04) (14) and Allpaths-LG (version r41043) (15). The final draft assembly contained 139 contigs in 139 scaffolds. The total size of the genome is 7.6 Mbp, and the final assembly is based on 932.3 Mbp of Illumina data, which provide an average 122.9× coverage of the genome.

The draft genome sequence of *Frankia* sp. BMG5.12 was resolved to 139 scaffolds consisting of 7,589,313 bp, with a G+C content of 71.67%, 6,253 candidate protein-coding genes, 51 tRNA genes, and 2 rRNA regions.

### Nucleotide sequence accession numbers.

The *Frankia* sp. BMG5.12 genome sequence has been deposited at DDBJ/EMBL/GenBank under the accession no. ARFH00000000. The version described in this paper is the first version, accession no. ARFH01000000.
